# Four Decades of Molecular Innovation in Chronic Myeloid Leukemia: From Antisense Targeting to Treatment-Free Remission

**DOI:** 10.3390/cancers18121922

**Published:** 2026-06-12

**Authors:** Maria Stefania De Propris, Alessandro Laganà, Massimo Breccia, Paolo De Fabritiis

**Affiliations:** 1 Hematology, Department of Translational and Precision Medicine, Az. Policlinico Umberto I-Sapienza University, 00161 Rome, Italy; depropris@bce.uniroma1.it (M.S.D.P.); lagana@bce.uniroma1.it (A.L.); breccia@bce.uniroma1.it (M.B.); 2 Department of Biomedicina e Prevenzione, Tor Vergata University, 00133 Rome, Italy

**Keywords:** chronic myeloid leukemia (CML), BCR::ABL1 oncogene, tyrosine kinase inhibitors (TKIs), treatment-free remission

## Abstract

Chronic myeloid leukemia (CML) represents a paradigm of targeted therapy, driven by the BCR::ABL1 fusion kinase. Over the past four decades, therapeutic strategies have evolved from early molecular targeting approaches and interferon-α to tyrosine kinase inhibitors (TKIs), dramatically improving survival and transforming CML into a largely controllable disease. To provide a comprehensive overview of this evolution, we conducted a narrative literature search across the PubMed and Embase databases, selecting peer-reviewed articles, international guidelines, and landmark clinical trials based on their historical and clinical relevance. Through this expert-driven synthesis, focusing on key milestones in CML therapy, including antisense strategies, interferon-based treatment, first-, second-, and third-generation TKIs, and the development of allosteric inhibitors, this paper analyzes current management strategies, treatment-free remission (TFR), and emerging therapies. The introduction of imatinib established proof of principle for oncogene-targeted therapy, leading to sustained survival improvements. Second- and third-generation TKIs further enhanced response depth and addressed resistance, including the T315I mutation. More recently, the development of the allosteric inhibitor asciminib introduced a novel mechanism of action and expanded therapeutic options for pretreated patients. Furthermore, the achievement of deep molecular responses has enabled TFR in approximately 40–60% of selected patients, redefining treatment goals toward functional cure. Emerging agents, including next-generation ATP-competitive and allosteric inhibitors, are showing promising activity in resistant disease and may further improve outcomes. Thus, CML represents a unique model of translational oncology, demonstrating how mechanistic insight can drive therapeutic innovation. Future strategies will focus on increasing TFR rates, overcoming resistance, targeting leukemic stem cells, and improving global access to therapy and monitoring, with the ultimate aim of achieving functional cure in the majority of patients.

## Simple Summary

Chronic myeloid leukemia (CML) represents one of the most successful examples of precision oncology. The identification of the BCR::ABL1 oncogene and the development of tyrosine kinase inhibitors (TKIs) have transformed CML from a fatal disease into a chronic condition with near-normal life expectancy. Over time, therapeutic strategies have evolved from early molecular targeting approaches and interferon-based treatments to increasingly potent TKIs and, more recently, to allosteric inhibitors such as asciminib. These advances have enabled deep molecular responses and introduced the concept of treatment-free remission (TFR), shifting the goal of therapy toward functional cure. This review summarizes four decades of therapeutic progress in CML, highlighting key milestones, current personalized management strategies, and emerging treatments. Ongoing challenges—including drug resistance, persistence of leukemic stem cells, and global disparities in access to care—are also discussed, together with future directions aimed at improving long-term outcomes and expanding the proportion of patients eligible for TFR.

## 1. Introduction

Chronic myeloid leukemia (CML) occupies a unique place in the history of modern oncology and has long served as a paradigm for molecularly targeted cancer therapy. It was the first human malignancy in which a recurrent chromosomal abnormality was identified as the causal event, the first in which the molecular architecture of this abnormality was elucidated, and ultimately the first disease in which pharmacologic inhibition of a single oncogenic driver produced durable disease control and near-normal life expectancy [1,2,3].

Globally, CML has an annual incidence of approximately 1.5 cases per 100,000 individuals and accounts for 10–15% of adult leukemias [3,4,5]. Over the past two decades, its epidemiology has been profoundly reshaped by therapeutic progress. Annual mortality has declined dramatically—from approximately 10–20% in the pre-imatinib era to around 1% with contemporary tyrosine kinase inhibitor (TKI) therapy [5,6]. As survival has improved, disease prevalence has steadily increased and is projected to exceed 180,000 patients in the United States alone by 2030 [5,7].

The cytogenetic hallmark of CML, the Philadelphia (Ph) chromosome, was first described by Nowell and Hungerford in 1960 [1]. In 1973, Rowley demonstrated that it results from a reciprocal translocation between chromosomes 9 and 22, t(9;22)(q34;q11) [8]. This rearrangement generates the BCR::ABL1 fusion gene, which encodes a constitutively active tyrosine kinase responsible for malignant transformation [9,10]. The causal role of this oncogene was definitively established in 1990 when Daley, Baltimore, and colleagues demonstrated that expression of BCR::ABL1 induces a CML-like myeloproliferative disorder in murine models [11,12].

**Figure 1:**
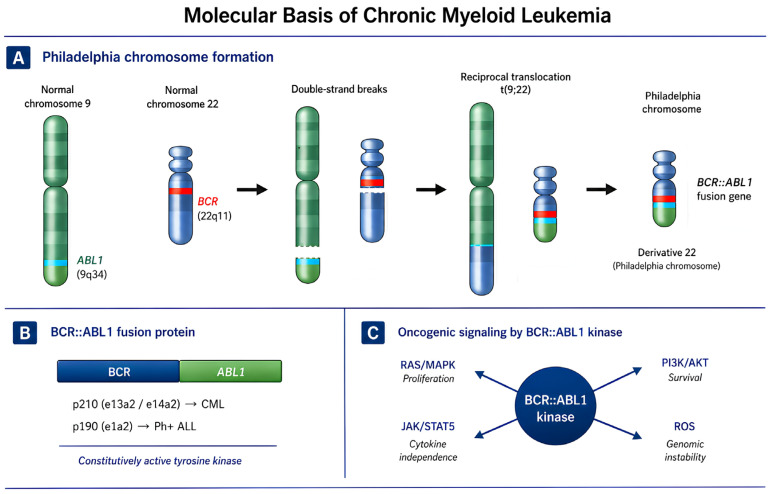
**Molecular Pathogenesis of Chronic Myeloid Leukemia.** (**A**) Formation of the Philadelphia chromosome through the reciprocal translocation t(9;22)(q34;q11). (**B**) Structure of the BCR::ABL1 fusion gene and major transcript variants. The p210 isoform is characteristic of CML, whereas p190 is more commonly associated with Ph-positive Acute Lymphoblastic Leukemia (Ph-pos ALL). (**C**) Constitutive BCR::ABL1 kinase activity activates key downstream pathways involved in leukemic cell proliferation, survival and genomic instability. ALL, acute lymphoblastic leukemia; CML, chronic myeloid leukemia; Ph, Philadelphia chromosome.

Among the major BCR::ABL1 isoforms, two are of major clinical relevance (Figure 1). The p210 isoform, generated by the e13a2 or e14a2 transcripts, is present in more than 95% of patients with CML and in approximately 25% of Philadelphia chromosome–positive (Ph+) acute lymphoblastic leukemia (ALL) cases [3,4,13]. The p190 isoform, resulting from the e1a2 transcript, predominates in Ph+ ALL (>70%) and displays higher intrinsic tyrosine kinase activity [13,14].

Constitutive BCR::ABL1 kinase activity perturbs multiple signaling networks in hematopoietic cells. Activation of the RAS/MAPK pathway promotes proliferation [15], stimulation of the PI3K/AKT pathway confers resistance to apoptosis [16], and activation of the JAK/STAT5 pathway drives cytokine independence and altered differentiation [17]. In addition, BCR::ABL1-induced reactive oxygen species promote genomic instability, facilitating clonal evolution and disease progression. Together, these mechanisms define the molecular pathogenesis of CML and provide the biological basis for targeted therapeutic strategies (Figure 1).

Long before the advent of tyrosine kinase inhibitors, CML also served as one of the earliest experimental platforms for molecularly targeted therapies directed at oncogenic transcripts. In the early 1990s, antisense oligodeoxynucleotides (AS-ODNs) targeting BCR::ABL1 mRNA were explored as a strategy to selectively suppress expression of the leukemogenic fusion gene. These studies demonstrated that inhibition of BCR::ABL1 expression could reduce proliferation and induce apoptosis in leukemic cells, providing early proof-of-concept that the disease might be controlled through direct molecular targeting of its primary oncogenic driver [18,19]. Although antisense approaches ultimately faced technical and pharmacologic limitations, they played a critical conceptual role in establishing BCR::ABL1 as a therapeutically actionable target and paving the way for subsequent kinase-directed drug development.

The introduction of the BCR::ABL1 tyrosine kinase inhibitor imatinib at the turn of the century transformed this conceptual framework into clinical reality, marking the beginning of the targeted therapy era in oncology [20,21,22]. Subsequent development of second- and third-generation TKIs further improved response depth and durability, dramatically reducing disease progression and establishing CML as one of the most successfully controlled malignancies in modern medicine.

More recently, the therapeutic goals of CML management have evolved beyond long-term disease control toward the possibility of treatment-free remission (TFR). Sustained deep molecular responses now allow a substantial proportion of patients to safely discontinue TKI therapy while maintaining durable remission, introducing a new paradigm in cancer therapy in which functional cure may be achieved without continuous treatment [23,24,25,26,27].

**Figure 2:**
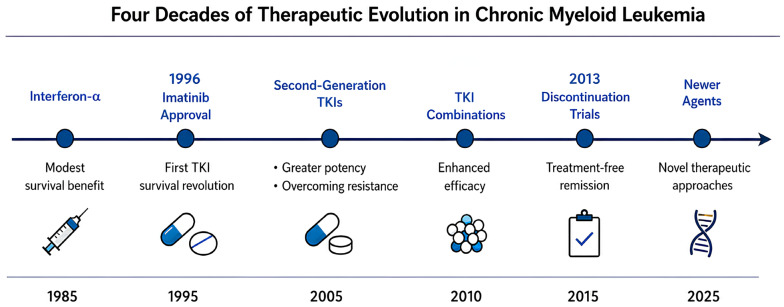
**Four Decades of Therapeutic Evolution in CML.** Timeline highlighting major milestones in CML treatment, from interferon-α and early molecular targeting approaches to the introduction of imatinib, second- and third-generation tyrosine kinase inhibitors (TKIs), Treatment-Free Remission (TFR) and emerging therapies including allosteric BCR::ABL1 inhibition.

In this review, we examine the therapeutic evolution of CML over nearly four decades, beginning with early molecular targeting strategies using antisense oligodeoxynucleotides, followed by the interferon-α era, the imatinib revolution, and the subsequent development of second- and third-generation TKIs. We also discuss the emergence of the allosteric STAMP (Specifically Targeting the ABL Myristoyl Pocket) inhibitor asciminib, the development of the treatment-free remission paradigm, and the parallel therapeutic advances in Philadelphia chromosome–positive acute lymphoblastic leukemia. Finally, we summarize the current landscape of CML management in 2026 and consider future directions for the field (Figure 2).

## 2. Early Molecular Targeting Strategies: The Antisense Era

Before the development of tyrosine kinase inhibitors, chronic myeloid leukemia already represented one of the earliest experimental models for molecularly targeted therapy. In the early 1990s, antisense oligodeoxynucleotides (AS-ODNs) directed against oncogenic transcripts were explored as a strategy to selectively inhibit gene expression at the mRNA level. The BCR::ABL1 fusion gene provided an especially attractive target for this approach, as the unique nucleotide sequence generated at the BCR–ABL1 junction is present exclusively in Philadelphia chromosome–positive leukemic cells and absent from normal hematopoietic tissues [18,28,29].

The first experimental evidence supporting this strategy was reported in 1991, when antisense oligodeoxynucleotides complementary to BCR::ABL1 mRNA were shown to inhibit proliferation and clonogenic growth of leukemic cells in vitro [29]. Shortly thereafter, in vivo studies confirmed that antisense-mediated suppression of BCR::ABL1 expression could impair leukemic cell survival and proliferation in experimental models [30].

At the same time, antisense approaches targeting other genes implicated in leukemic cell proliferation were also investigated. In particular, suppression of the transcription factor c-myb, a key regulator of hematopoietic progenitor self-renewal, was shown to selectively inhibit leukemic colony formation while sparing normal hematopoietic progenitors. These studies provided early proof-of-concept that antisense strategies could be exploited to selectively eliminate leukemic cells while preserving normal hematopoiesis [28].

Subsequent work translated these observations into clinically oriented studies. Antisense oligodeoxynucleotides directed against the BCR::ABL1 junction were shown to selectively reduce the number of Philadelphia chromosome–positive colonies in autologous bone marrow samples while maintaining normal progenitor activity, establishing the feasibility of an ex vivo purging strategy aimed at improving autologous transplantation outcomes [19]. Further investigations confirmed the sequence-specific inhibition of CML clonogenic growth by BCR-ABL–targeted oligodeoxynucleotides across multiple patient-derived samples [31]. These experimental findings ultimately led to the first clinical protocol evaluating antisense-mediated purging of leukemic cells in patients with advanced-phase CML undergoing autologous transplantation [32].

Although antisense oligodeoxynucleotide approaches were limited by pharmacologic challenges—including inefficient intracellular delivery, nuclease degradation, and insufficient in vivo potency—they played an important conceptual role in the evolution of CML therapy. These studies provided some of the earliest demonstrations that direct molecular targeting of the BCR::ABL1 oncogenic driver could suppress leukemic cell growth, thereby establishing the biological validity of BCR::ABL1 as a therapeutic target.

Retrospectively, the antisense era represents a formative stage in the development of targeted therapy for CML. While the clinical impact of antisense oligodeoxynucleotides remained limited, the central concept they embodied—that the unique molecular lesion driving the disease could be selectively targeted—proved fundamentally correct. The subsequent success of BCR::ABL1 kinase inhibitors therefore reflected not a change in therapeutic rationale but rather the emergence of pharmacologic tools capable of fully exploiting the molecular vulnerability that these early studies had already identified [2,3,18].

## 3. The Interferon-α Era: The First Disease-Modifying Therapy in CML

Before the advent of tyrosine kinase inhibitors, the first therapy capable of significantly altering the natural history of chronic myeloid leukemia was interferon-α (IFN-α). Introduced in the 1980s, IFN-α represented the earliest pharmacologic approach able to induce durable hematologic and cytogenetic responses in a substantial proportion of patients with chronic-phase CML [33].

Initial clinical studies demonstrated that IFN-α therapy could achieve complete hematologic remission in 60–80% of patients and partial or complete cytogenetic responses in approximately 20–30%, outcomes that were previously unattainable with conventional cytoreductive agents such as busulfan or hydroxyurea. Importantly, patients achieving cytogenetic responses experienced significantly improved long-term survival, establishing cytogenetic response as a key therapeutic endpoint in CML management [34,35,36].

The mechanisms underlying the clinical activity of interferon-α were complex and multifactorial. IFN-α exerts direct antiproliferative effects on leukemic progenitors, promotes differentiation, and enhances immune-mediated control of malignant clones through activation of cytotoxic T cells and natural killer cells. In addition, interferon signaling modulates the bone marrow microenvironment and may preferentially suppress Philadelphia chromosome–positive progenitors, thereby allowing residual normal hematopoiesis to re-emerge.

Although IFN-α rarely eradicated the leukemic clone, it represented the first therapy capable of modifying the natural history of the disease and significantly prolonging survival in CML. Large cooperative group studies conducted in the 1990s demonstrated that patients treated with interferon-α had markedly improved long-term outcomes compared with those receiving conventional chemotherapy [36,37].

By the late 1990s, interferon-α had therefore become the standard first-line therapy for chronic-phase CML. However, treatment was frequently associated with substantial toxicity—including flu-like symptoms, fatigue, depression, and autoimmune complications—and only a minority of patients achieved deep or durable cytogenetic responses. These limitations underscored the need for more selective and effective therapies targeting the molecular driver of the disease.

The identification of BCR::ABL1 kinase activity as the central pathogenic event in CML, together with accumulating experimental evidence that inhibition of this oncogenic signal could suppress leukemic growth, ultimately led to the development of small-molecule tyrosine kinase inhibitors. The clinical introduction of imatinib at the turn of the century would soon transform CML from a chronic fatal disease into the prototype of successful targeted cancer therapy.

## 4. The Imatinib Revolution: Proof of Principle for Targeted Therapy

### 4.1. Discovery and Mechanism of Action

The development of imatinib mesylate (STI571; Gleevec/Glivec) represents a landmark achievement in translational medicine and rational drug design [2,3,38]. Its development was enabled by the progressive elucidation of ABL kinase structure and function, together with the demonstration that constitutive BCR::ABL1 kinase activity is both necessary and sufficient to drive the CML phenotype [10,11].

A medicinal chemistry program at Ciba-Geigy (later Novartis) screened 2-phenylaminopyrimidine derivatives to identify selective protein kinase inhibitors, ultimately leading to the identification of STI571 as a potent ABL inhibitor [38]. Preclinical studies by Druker and colleagues demonstrated that imatinib selectively inhibited proliferation of BCR::ABL1-positive cells while sparing normal hematopoietic progenitors, providing a strong therapeutic rationale [2].

Structural studies subsequently clarified the molecular basis of this selectivity. Imatinib binds the ATP-binding cleft of the ABL kinase domain, stabilizing the enzyme in its inactive (“DFG-out”) conformation and thereby preventing substrate phosphorylation [39,40]. This requirement for a specific inactive conformation restricts its activity to a limited set of kinases—including ABL, KIT, and PDGFR—contributing to its favorable therapeutic index.

Early clinical development rapidly confirmed these expectations. In the landmark phase I trial initiated in 1998, imatinib induced complete hematologic responses in nearly all patients with chronic-phase CML resistant to interferon-α, and complete cytogenetic responses (CCyR) in more than half of patients treated at doses ≥300 mg/day [20]. These results were unprecedented and signaled a profound shift in therapeutic possibilities.

### 4.2. Clinical Outcomes: A Paradigm Shift

The transformative impact of imatinib was definitively established by the IRIS (International Randomized Study of Interferon and STI571) trial, which compared imatinib 400 mg daily with interferon-α plus low-dose cytarabine in newly diagnosed chronic-phase CML [21].

At 18 months, imatinib achieved a complete hematologic response rate of 98%, a CCyR rate of 76%, and a major molecular response (MMR) rate of approximately 39%, all significantly superior to the comparator arm. Importantly, responses continued to deepen with prolonged therapy, with the majority of patients ultimately achieving durable cytogenetic and molecular remissions.

Long-term follow-up of the IRIS cohort confirmed the durability of these outcomes. At 5 years, overall survival approached 90% [22], and subsequent analyses demonstrated sustained disease control with low rates of progression to advanced phases [41]. The 10-year follow-up reported an overall survival of approximately 83%, with CML-related mortality reduced to about 1% per year [6].

These results fundamentally altered the natural history of CML. What had previously been a fatal disease with a median survival of 3–5 years became a chronic condition compatible with near-normal life expectancy. More broadly, imatinib provided the first definitive clinical proof that selective inhibition of a single oncogenic driver could produce durable disease control in cancer, establishing a paradigm that would reshape oncology.

Subsequent studies, including the German CML Study IV, confirmed that imatinib 400 mg daily represents the optimal frontline dose, as higher doses accelerated early molecular responses but did not translate into superior long-term survival outcomes [42].

### 4.3. Emergence of Resistance

Despite its remarkable efficacy, a subset of patients exhibited either primary resistance (failure to achieve response milestones) or secondary resistance (loss of a previously achieved response). Overall, resistance or intolerance was observed in approximately 15–25% of patients over long-term follow-up [22,43].

The best-characterized mechanism of resistance involves point mutations within the BCR::ABL1 kinase domain. Gorre et al. first demonstrated in 2001 that such mutations, as well as gene amplification, could mediate clinical resistance to imatinib [44]. Subsequent studies identified a broad spectrum of mutations with differential sensitivity to TKIs, forming the basis for mutation-guided therapeutic strategies [45].

Among these, the T315I “gatekeeper” mutation is particularly clinically relevant. By substituting threonine with isoleucine at position 315, this mutation disrupts a critical hydrogen bond required for binding of imatinib and most second-generation TKIs, conferring high-level resistance [40,46].

In addition to kinase domain mutations, several BCR::ABL1-independent mechanisms contribute to resistance. These include altered drug transport—such as reduced activity of the OCT-1 influx transporter or increased expression of efflux pumps like MDR1—as well as activation of alternative signaling pathways, including SRC family kinases (e.g., LYN), PI3K/AKT, and Wnt/β-catenin signaling [47,48,49]. Leukemic stem cells (LSCs) represent a central component of this BCR::ABL1-independent resistance. These cells are relatively insensitive to TKIs due to their quiescent state, metabolic adaptation, and reliance on alternative survival programs, including Wnt/β-catenin and BCL-2–related pathways. As a result, they persist despite effective kinase inhibition and constitute a major barrier to disease eradication. Collectively, these observations indicate that resistance in CML is not solely a consequence of impaired drug binding to BCR::ABL1 but reflects a broader, multi-layered process involving both molecular alterations and stem cell–driven persistence [50]. The recognition of these mechanisms has directly informed the development of next-generation and mechanistically distinct therapeutic strategies.

## 5. Second- and Third-Generation TKIs: Overcoming Resistance and Optimizing Therapy

The emergence of imatinib resistance and the need for deeper and faster responses led to the development of second-generation TKIs, including dasatinib, nilotinib, and bosutinib (Table 1).

Dasatinib is a dual SRC/ABL inhibitor with the unique ability to bind both active and inactive conformations of the ABL kinase, resulting in greater potency and broader activity against imatinib-resistant mutations [50,51,52]. Clinical trials demonstrated robust efficacy in patients with imatinib-resistant or -intolerant disease.

Nilotinib, a structurally modified derivative of imatinib, exhibits higher binding affinity for the ATP-binding site and approximately 20–30-fold greater potency in vitro [53]. It demonstrated significant clinical activity in resistant disease and was subsequently evaluated as frontline therapy.

Bosutinib, another dual SRC/ABL inhibitor with minimal activity against KIT and PDGFR, offers a distinct toxicity profile. In the BELA trial, bosutinib achieved higher rates of major molecular response compared with imatinib, although early cytogenetic response rates were similar [54].

Randomized frontline studies (DASISION, ENESTnd, BFORE) demonstrated that second-generation TKIs induce faster and deeper molecular responses than imatinib, leading to their adoption as frontline options in selected patients, particularly those with higher-risk disease or where early deep molecular response is a priority.

Olverembatinib (HQP1351) is a third-generation TKI developed in China with potent activity against both native BCR::ABL1 and the T315I mutation. Clinical studies have demonstrated high rates of cytogenetic and molecular responses in patients resistant or intolerant to prior TKIs, including those harboring T315I, with a manageable safety profile and a relatively low incidence of cardiovascular events. Approved in China, olverembatinib is currently being evaluated in global studies and may expand therapeutic options for patients with resistant disease [55,56].

### 5.1. The T315I Challenge and Ponatinib

The T315I mutation represented the most significant therapeutic barrier in the TKI era. Ponatinib, a third-generation TKI, was specifically designed to overcome this mutation through a unique structural modification that avoids steric hindrance at the gatekeeper residue [55].

In the phase II PACE trial, ponatinib demonstrated major cytogenetic responses in approximately 54% of patients with chronic-phase CML and in up to 70% of those harboring the T315I mutation [55]. Long-term follow-up confirmed the durability of these responses [57]. However, ponatinib use was complicated by an increased incidence of arterial occlusive events (AOEs), which emerged as a major safety concern. Rather than permanent withdrawal, regulatory agencies implemented risk mitigation strategies, including dose optimization and careful patient selection.

The OPTIC trial subsequently established a response-based dosing strategy—starting at 45 mg daily with reduction upon achieving BCR::ABL1 ≤ 1% (IS)—which significantly reduced vascular toxicity while maintaining efficacy. This approach has become the standard for ponatinib administration in clinical practice.

### 5.2. Molecular Monitoring and Treatment Milestones

The success of TKI therapy necessitated the development of standardized response criteria and monitoring strategies. The European LeukemiaNet (ELN) has provided iterative recommendations (2009, 2013, 2020, 2025), defining response milestones that guide therapeutic decisions [58].

Current ELN criteria define:

- Optimal response: BCR::ABL1 transcript levels ≤10% (3 months), ≤1% (6 months), and ≤0.1% (MMR) at 12 months.
- Warning: intermediate values requiring closer monitoring.
- Failure: lack of hematologic or cytogenetic response at defined time points (Table 2).

Quantitative real-time PCR standardized on the International Scale (IS) remains the cornerstone of disease monitoring. Assessments are performed every 3 months during the first 2 years and every 3–6 months thereafter in patients with stable responses. The introduction of standardized molecular monitoring not only enabled early identification of treatment failure but also laid the foundation for the concept of deep molecular response (MR^4^, MR^4.5^), which would later become central to treatment-free remission strategies.

## 6. Allosteric Targeting and the Treatment-Free Remission Era: Toward Functional Cure

### 6.1. Asciminib and the STAMP Paradigm: Targeting the Myristoyl Pocket

While second- and third-generation ATP-competitive TKIs significantly improved outcomes in CML, they did not fully overcome two major limitations: the persistence of leukemic stem cells and the emergence of resistance, particularly in heavily pretreated patients. These challenges prompted the exploration of alternative strategies capable of targeting BCR::ABL1 through mechanisms distinct from ATP-competitive inhibition.

Asciminib (ABL001) represents the first-in-class inhibitor of the STAMP (Specifically Targeting the ABL Myristoyl Pocket) paradigm and marks a conceptual shift in kinase targeting [59,60]. Unlike all prior TKIs, asciminib binds to the myristoyl pocket of the ABL kinase domain—a regulatory site normally engaged by the N-terminal myristoylated cap in the native ABL protein.

In BCR::ABL1, the fusion event removes this autoregulatory mechanism, resulting in constitutive kinase activation. Asciminib restores this physiological inhibition by stabilizing the kinase in an inactive conformation, effectively mimicking the natural autoinhibitory state. This allosteric mechanism is fundamentally distinct from ATP-competitive inhibition and allows for highly selective targeting of BCR::ABL1 with minimal off-target activity.

Importantly, because asciminib does not bind the ATP site, it retains activity against many kinase domain mutations that confer resistance to conventional TKIs. Even in the presence of T315I, asciminib demonstrates clinically meaningful activity at increased doses, although resistance can still emerge through mutations in the myristoyl pocket or compound mutation profiles.

**Figure 3:**
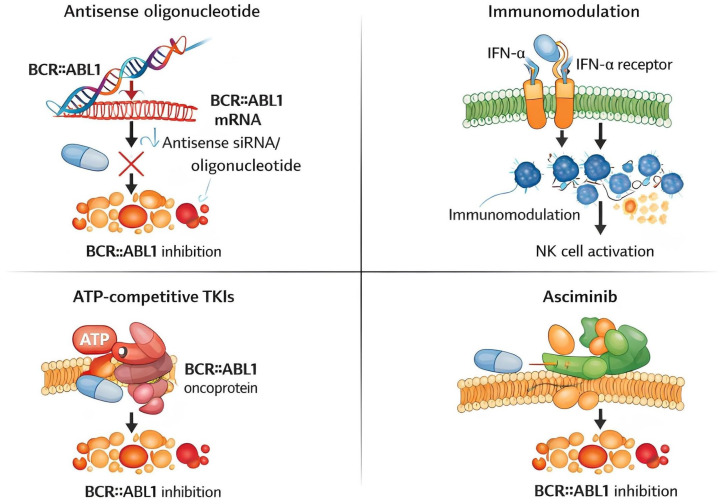
**Mechanisms of Action of Targeted Therapies.** Comparison of different molecular strategies: Antisense oligonucleotides targeting BCR::ABL1 mRNA. Interferon-α providing immunomodulatory and antiproliferative effects; ATP-competitive TKIs (e.g., imatinib, dasatinib, nilotinib) binding the catalytic site. Asciminib, a first-in-class STAMP inhibitor that restores physiological autoinhibition by binding the ABL myristoyl pocket. IFN-α, interferon alpha, TKI, tyrosine kinase inhibitor.

The development of asciminib therefore represents not only a new agent but the introduction of an entirely new therapeutic class, introducing allosteric inhibition as a viable strategy in oncology (Figure 3). More broadly, the emergence of allosteric ABL inhibitors has expanded the therapeutic landscape of CML beyond ATP-competitive TKIs. By targeting a regulatory site distinct from the ATP-binding pocket, asciminib provides a complementary mechanism of inhibition characterized by high selectivity and activity against many ATP-site mutations. This has generated a strong rationale for combination approaches, in which simultaneous targeting of the ATP-binding site and the myristoyl pocket may enhance pathway suppression and reduce the likelihood of resistant clone emergence [60]. The success of asciminib has also renewed interest in the development of additional allosteric inhibitors and rational combination strategies aimed at overcoming compound resistance and achieving deeper molecular responses. These advances suggest that allosteric targeting may become an increasingly important component of future CML therapy.

### 6.2. Clinical Development and Positioning of Asciminib

The clinical activity of asciminib was first demonstrated in heavily pretreated patients with CML. In early-phase trials, asciminib achieved substantial rates of major molecular response (MMR) in patients who had failed multiple prior TKIs, including those harboring T315I mutations [59].

The pivotal phase III ASCEMBL trial compared asciminib with bosutinib in patients with chronic-phase CML previously treated with at least two TKIs. Asciminib demonstrated superior efficacy, with significantly higher MMR rates at 24 weeks (25.5% vs. 13.2%), along with a more favorable tolerability profile [61]. These results led to its approval as a standard option in later-line therapy.

Subsequent studies have explored asciminib in earlier lines of treatment and in combination with ATP-competitive TKIs. The rationale for combination therapy is particularly compelling: simultaneous targeting of the ATP-binding site and the myristoyl pocket may produce synergistic inhibition and reduce the likelihood of resistance through compound mutations.

Beyond efficacy, asciminib is characterized by a distinct safety profile, with lower rates of cardiovascular toxicity compared with some second- and third-generation TKIs. This feature may become increasingly relevant as long-term survivorship and treatment discontinuation strategies gain importance.

Taken together, asciminib expands the therapeutic landscape of CML and provides a mechanistically complementary approach to ATP-competitive inhibition.

The success of asciminib has stimulated the development of additional allosteric and highly selective ABL1 inhibitors. ELVN-001 is a selective active-site inhibitor designed to retain activity across a broad spectrum of BCR::ABL1 resistance mutations, as those rendering second-generation TKIs ineffective (including the T315I); early-phase trial data have demonstrated safety and molecular responses in heavily pretreated patients [62]. TERN-701 and TGRX-678 are novel allosteric STAMP inhibitors targeting the myristoyl pocket, with preliminary studies demonstrating substantial molecular response rates, including in patients with prior TKI or even prior STAMP inhibitor exposure, owing to their high potency and distinct pharmacokinetic profiles [63,64]. Crucially, these allosteric alternatives offer the potential for combination strategies with ATP-competitive inhibitors to prevent the emergence of compound mutations. Collectively, these emerging agents highlight a rapidly evolving therapeutic landscape and support the potential of both ATP-site and allosteric strategies to overcome resistance and deepen molecular responses in CML.

### 6.3. Deep Molecular Response and the Emergence of Treatment-Free Remission

The progressive refinement of TKI therapy has led to increasingly deep molecular responses, quantified as MR^4^ (BCR::ABL1 ≤ 0.01% on the IS), MR^4.5^ (≤0.0032% on the IS with ≥32.000 ABL1 copies), and MR^5^ (≤0.001% IS with ≥100.000 ABL1 copies) [65]. These deep responses provided the biological foundation for a paradigm shift in CML management: the possibility of treatment-free remission (TFR). This represents a transition from continuous oncogene suppression to a functional cure, demonstrating that a subset of patients can maintain long-term disease control without ongoing therapy.

The first prospective evidence supporting TFR came from the STIM (Stop Imatinib) trial, which demonstrated that approximately 40% of patients with sustained deep molecular response could discontinue imatinib without molecular relapse [66]. Subsequent studies—including EURO-SKI, ENESTfreedom, and DASFREE—confirmed that TFR is reproducibly achievable across different TKIs, with long-term remission rates of 40–60% in carefully selected patients (Table 3) [23,24,67].

**Figure 4:**
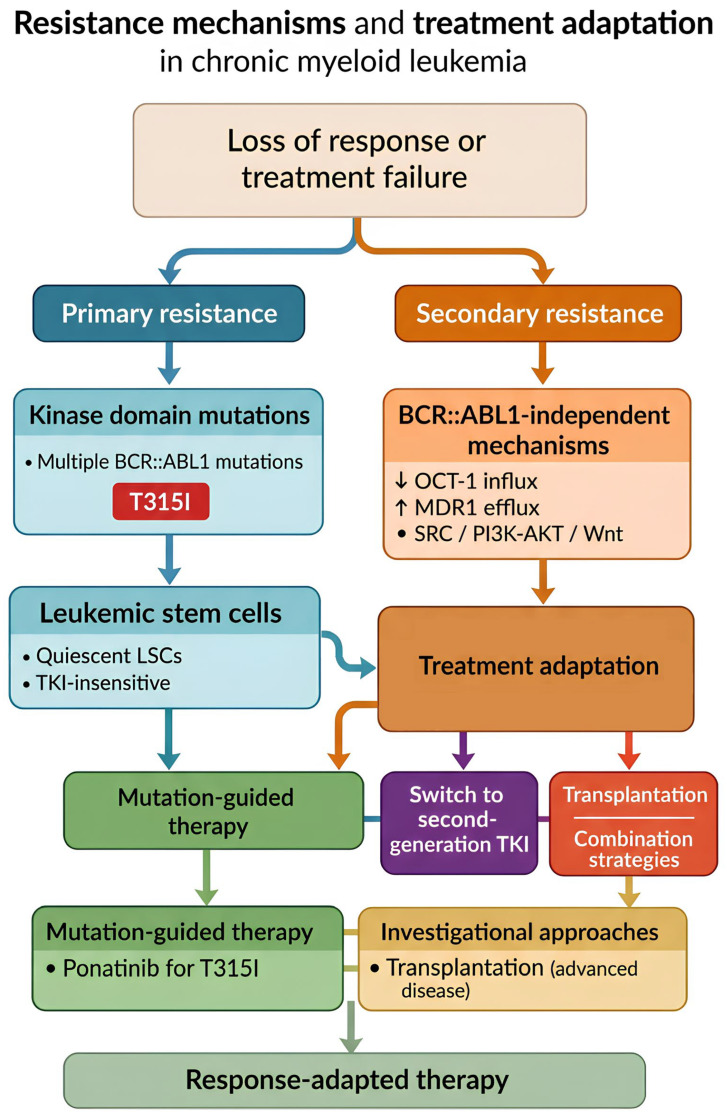
**Resistance Mechanisms and Clinical Adaptation.** Flowchart of primary and secondary resistance to TKI therapy. Key mechanisms include kinase domain mutations (e.g., the T315I “gatekeeper” mutation), BCR::ABL1-independent pathway activation, and leukemic stem cell (LSC) persistence. The figure outlines response-adapted strategies, including mutation-guided TKI selection (e.g., ponatinib for T315I) and the role of allogeneic stem cell transplantation in advanced phases. TKI, tyrosine kinase inhibitor.

A consistent observation across studies is that most molecular relapses occur within the first 6 months after discontinuation, and that re-initiation of TKI therapy leads to rapid regain of molecular response in the vast majority of patients. This has established TFR as a safe and clinically viable strategy when conducted under strict molecular monitoring (Figure 4).

Predictors of successful TFR include longer duration of TKI therapy, sustained deep molecular response, low Sokal risk score, and possibly immunological factors. The latter observation has revived interest in the role of immune surveillance, specifically involving natural killer (NK) cells and T-cell-mediated responses, which play a crucial role in controlling residual leukemic stem cells and preventing clinical relapse after drug withdrawal. (Table 4). In real-world populations, optimizing patient selection prior to TKI discontinuation is crucial for both clinical outcomes and resource allocation. Recently, two independent study groups developed prognostic scores to predict successful TFR and improve candidate selection. Nevertheless, both groups underscored that the duration of stable DMR before TKI discontinuation remains the most critical independent prognostic factor [68,69].

### 6.4. Biological Basis of Treatment-Free Remission: Leukemic Stem Cells as the Next Therapeutic Frontier

Despite the remarkable success of tyrosine kinase inhibitors (TKIs), leukemic stem cells (LSCs) persist in the majority of patients with chronic myeloid leukemia (CML) and represent the principal biological barrier to definitive cure. In contrast to rapidly proliferating leukemic progenitors, CML LSCs are largely insensitive to BCR::ABL1 kinase inhibition because of their quiescent state and their dependence on survival programs that are only partially reliant on BCR::ABL1 signaling. As a consequence, residual LSCs may survive even in patients who achieve sustained deep molecular responses [50].

Over the last decade, increasing efforts have therefore focused on identifying the molecular and metabolic mechanisms responsible for LSC persistence. Multiple signaling pathways have been implicated, including aberrant activation of Wnt/β-catenin, Hedgehog, and JAK/STAT signaling, as well as dysregulation of autophagy, mitochondrial oxidative phosphorylation, and anti-apoptotic BCL-2 family proteins. These discoveries have opened new therapeutic perspectives aimed at selectively targeting the stem cell compartment beyond conventional kinase inhibition.

Several combinatorial strategies are currently under investigation. These include the association of TKIs with BCL-2 inhibitors such as venetoclax, interferon-α, immune-modulating agents, epigenetic therapies, and metabolic inhibitors designed to disrupt LSC energy dependence and survival. The rationale underlying these approaches is not only to deepen molecular responses but also to eradicate or functionally suppress the residual leukemic stem cell pool that sustains disease persistence.

At the same time, growing evidence suggests that immunological control plays a central role in successful treatment-free remission (TFR). Patients maintaining remission after TKI discontinuation frequently exhibit enhanced immune surveillance, including increased natural killer (NK)-cell activity, more effective cytotoxic T-cell responses, and favorable modulation of the bone marrow microenvironment. These observations support the concept that durable remission may depend on a dynamic equilibrium between residual leukemic cells and host immune control rather than on complete eradication of every malignant clone.

Collectively, these findings are reshaping the therapeutic paradigm of CML. Whereas the initial goal of therapy was hematologic and cytogenetic control, and later deep molecular remission, the emerging frontier is now the achievement of a durable functional cure: sustained disease control in the absence of continuous therapy. In this evolving scenario, future CML treatment strategies will likely integrate potent kinase inhibition with stem cell-directed and immune-based approaches specifically designed to eliminate or permanently restrain leukemic stem cells.

### 6.5. Integrating Allosteric Inhibition and TFR: Future Directions

The convergence of allosteric inhibition and treatment discontinuation strategies represents the current frontier in CML research.

Asciminib-based combinations may enhance depth of molecular response and accelerate achievement of MR^4 or deeper, thereby increasing the proportion of patients eligible for TFR. Early clinical studies exploring asciminib in combination with ATP-competitive TKIs suggest the potential for deeper and more rapid molecular responses, although long-term data are still emerging.

In parallel, novel therapeutic approaches aim to target leukemic stem cells more directly, including inhibitors of the Wnt/β-catenin pathway, BCL-2 family proteins, and immune-based strategies such as vaccines and checkpoint modulation. The integration of these approaches raises the possibility that TFR rates could be substantially increased, moving the field closer to a true functional cure for the majority of patients.

The evolution of CML therapy—from early antisense strategies to interferon-α, ATP-competitive TKIs, and now allosteric inhibition and treatment discontinuation—represents one of the most complete translational arcs in modern oncology. What began as an effort to suppress a single oncogenic transcript has culminated in a therapeutic paradigm in which long-term remission can be maintained in the absence of continuous treatment. CML thus stands not only as the prototype of targeted therapy but increasingly as a model for achieving functional cure in cancer.

## 7. Current Management of CML in 2026: An Integrated and Personalized Approach

The management of chronic myeloid leukemia in 2026 reflects the convergence of three decades of biological insight and therapeutic innovation. What was once a uniformly fatal disease is now a highly controllable condition, and in a substantial proportion of patients, a model of functional cure. Contemporary management is characterized by a personalized, response-adapted strategy, integrating disease biology, patient-specific factors, and long-term therapeutic goals.

### 7.1. Therapeutic Landscape and First-Line Selection

The current therapeutic armamentarium for chronic-phase CML includes multiple BCR::ABL1 inhibitors: generic imatinib, second-generation TKIs (dasatinib, nilotinib, bosutinib), and the allosteric inhibitor asciminib, which is increasingly incorporated into treatment algorithms as regulatory approvals expand across regions [5,58].

International guidelines, including those from the European LeukemiaNet [58] and NCCN, recommend that frontline TKI selection be individualized based on a combination of disease- and patient-related factors. These include:

- Disease risk scores (e.g., Sokal, ELTS).
- Comorbidities, particularly cardiovascular and pulmonary risk.
- Age and fitness.
- Potential drug–drug interactions.
- Patient preferences and treatment goals, including the desire to achieve treatment-free remission (TFR).

Second-generation TKIs are often favored in younger patients and in those with intermediate- or high-risk disease, due to their ability to induce faster and deeper molecular responses, thereby increasing the likelihood of achieving TFR eligibility. In contrast, imatinib remains an appropriate and widely used frontline option, particularly in older patients or those with significant comorbidities, owing to its well-established safety profile and long-term tolerability.

Bosutinib represents an alternative in patients with cardiovascular risk, while nilotinib and dasatinib require careful patient selection due to their association with vascular events and pleuro-pulmonary toxicity, respectively.

Recently, asciminib received FDA approval for newly diagnosed Ph+ CML-CP based on the landmark ASC4FIRST trial. In the frontline setting, asciminib demonstrated optimal rates of MMR achievement and a significantly better tolerability profile compared with investigator-selected TKIs, resulting in fewer dose modifications, a lower incidence of grade ≥3 adverse events (AEs), and a reduced rate of treatment discontinuation due to toxicity [70,71]. While clinically approved and used in China, olverembatinib is currently under investigation and has not yet been approved by the FDA and/or EMA [55].

### 7.2. Molecular Monitoring and Response-Adapted Management

Quantitative real-time PCR monitoring of BCR::ABL1 transcripts on the International Scale (IS) remains the cornerstone of CML management. Molecular assessments are performed every 3 months during the first two years of therapy and every 3–6 months thereafter in patients with stable responses [58,72].

Treatment decisions are guided by ELN-defined response milestones, which stratify patients into optimal response, warning, or failure categories. Early molecular response—particularly achieving BCR::ABL1 ≤10% at 3 months—has emerged as a critical predictor of long-term outcomes.

Failure to meet response milestones prompts therapeutic intervention, including dose adjustment, switching to an alternative TKI, or mutation analysis to guide subsequent therapy [73]. This dynamic, response-adapted approach represents one of the defining features of modern CML management.

### 7.3. Special Clinical Scenarios

#### 7.3.1. Elderly and Cardiovascular Risk in Comorbid Patients

In older patients (≥70 years) or those with significant comorbidities, treatment goals often prioritize tolerability and quality of life over rapid achievement of deep molecular responses. In this setting, imatinib remains a preferred option, although dose-adjusted second-generation TKIs or asciminib may be considered in selected cases. Given the association of certain TKIs—particularly nilotinib and ponatinib—with arterial occlusive events, cardiovascular risk stratification is essential. In high-risk patients, imatinib or bosutinib are generally preferred.

#### 7.3.2. Pregnancy

All TKIs are potentially teratogenic and are contraindicated, particularly during the first trimester. Interferon-α remains the preferred therapeutic option during pregnancy due to its favorable safety profile, necessitating careful preconception planning and alternative management strategies. Key aspects of CML pregnancy management include the timing of TKI discontinuation, the feasibility of treatment-free remission, and the role of alternative therapies such as interferon alpha. Additionally, it is mandatory to discuss with the patient the possibility of restarting treatment during pregnancy, the TKI selection in subsequent trimesters, and postpartum disease management, including breastfeeding considerations [74].

#### 7.3.3. Pediatric CML

Pediatric CML is rare but presents unique biological and clinical challenges. Pediatric CML cases may present unique molecular characteristics, such as dysregulation of the Rho pathway, which may contribute to clinical differences between pediatric and adult patients [75].

Additionally, children tend to have a more aggressive clinical presentation and, although the overall prognosis has dramatically improved in the TKI era, the management of pediatric CML requires particular attention to long-term toxicity, growth and developmental issues, treatment adherence, and quality of life [76]. Indeed, while TFR has become a therapeutic goal in adults in chronic phase with optimal response to therapy, data are currently insufficient to support TKI discontinuation in pediatrics outside of a clinical trial [77].

Imatinib remains the standard frontline therapy, with second-generation TKIs reserved for resistant or intolerant cases. Long-term TKI exposure in children has been associated with growth deceleration, altered bone metabolism, endocrine dysfunction, and potential effects on pubertal development, necessitating careful longitudinal monitoring.

#### 7.3.4. Advanced-Phase CML

Accelerated phase and blast crisis CML remain clinically challenging and are associated with inferior outcomes. Management typically involves:

- Use of potent TKIs (e.g., ponatinib, dasatinib, or asciminib in selected settings).
- Combination with chemotherapy in blast crisis.
- Early consideration of allogeneic stem cell transplantation, which remains the only established curative option in advanced disease.

Despite therapeutic advances, prevention of progression through optimal management in chronic phase remains the most effective strategy.

### 7.4. Global Access and Health Equity

One of the most pressing challenges in contemporary CML management is the disparity in global access to therapy and monitoring.

The availability of generic imatinib since patent expiration has dramatically improved access in low- and middle-income countries (LMICs), where annual treatment costs have decreased to a few hundred dollars. This has enabled large-scale treatment programs and significantly improved outcomes in resource-limited settings. However, access to second-generation TKIs and newer agents such as asciminib remains limited due to high costs, often exceeding $30,000–100,000 per year. In addition, the lack of standardized molecular monitoring infrastructure in many regions hampers optimal response assessment and limits the implementation of TFR strategies.

These disparities underscore a critical ethical dimension of modern CML care: while the disease is now highly manageable—and in some cases functionally curable—the benefits of modern therapy are not yet equitably distributed worldwide.

In 2026, the management of CML exemplifies the successful integration of molecular biology, targeted therapy, and personalized medicine. Yet, important challenges remain, including the eradication of leukemic stem cells, the optimization of treatment discontinuation strategies, and the reduction in global disparities in access to care.

## 8. Conclusions

Over the past four decades, chronic myeloid leukemia has evolved from a uniformly fatal hematologic malignancy into one of the most compelling success stories in modern oncology. This transformation reflects a unique convergence of fundamental biological discovery, translational research, and rational drug development. From the early identification of the Philadelphia chromosome to the molecular dissection of the BCR::ABL1 oncogene, and from the first antisense strategies to the development of highly effective tyrosine kinase inhibitors, CML has consistently served as a paradigm for precision medicine.

Importantly, the trajectory of therapeutic innovation in CML has not been linear but cumulative. Early experimental approaches—such as antisense oligodeoxynucleotides—played a critical conceptual role by demonstrating that selective targeting of the leukemogenic driver was feasible. This foundational insight ultimately enabled the development of BCR::ABL1 kinase inhibitors, which transformed disease outcomes and redefined the natural history of CML. The subsequent refinement of TKI therapy, including second- and third-generation inhibitors and, more recently, the introduction of allosteric agents such as asciminib, has further expanded therapeutic possibilities and addressed key mechanisms of resistance.

**Figure 5:**
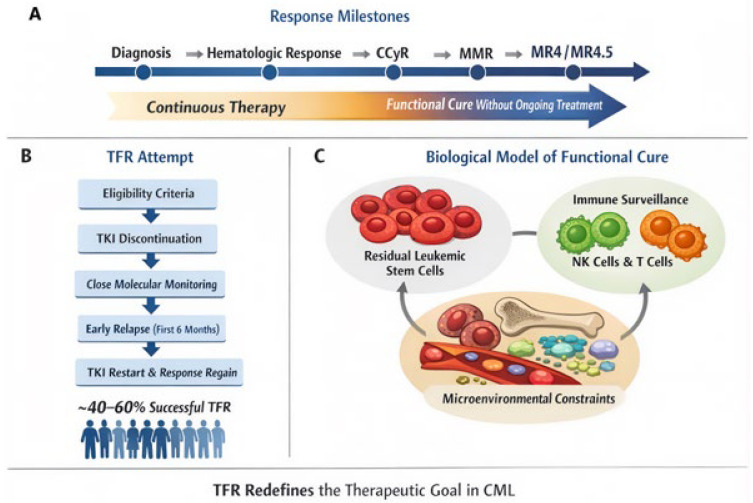
**The Paradigm of Treatment-Free Remission (TFR).** (**A**) Response milestones required for TFR eligibility, emphasizing sustained deep molecular response (MR4/MR4.5). (**B**) Clinical pathway for TKI discontinuation under strict molecular monitoring. (**C**) Biological model of functional cure: TFR is maintained through a combination of immune surveillance (NK and T cells) and microenvironmental constraints that keep residual leukemic stem cells under control despite the absence of therapy. CCyR, complete cytogenetic response; MMR, major molecular response; MR, molecular response; TFR, treatment-free remission; TKI, tyrosine kinase inhibitor.

Perhaps the most profound shift in recent years has been the transition from lifelong disease control to the pursuit of treatment-free remission (TFR). The ability to safely discontinue therapy in a substantial proportion of patients while maintaining durable remission represents a conceptual breakthrough, challenging the traditional paradigm that continuous treatment is required to suppress malignant disease. In this context, CML has become not only a model of targeted therapy but also a prototype of functional cure and a cumulative model of translational oncology (Figure 5).

Despite these remarkable advances, important challenges remain. Leukemic stem cells persist in most patients and continue to represent a barrier to definitive eradication. An important unresolved question is whether durable TFR truly reflects stable immunological equilibrium or merely prolonged suppression of residual leukemic stem cells below the threshold of clinical detection. Increasing evidence suggests that leukemic stem cells survive independently of BCR::ABL1 kinase activity through activation of alternative pathways involving Wnt/β-catenin, Hedgehog signaling, autophagy, mitochondrial metabolism, and anti-apoptotic BCL-2 family proteins. These observations may explain why complete molecular eradication remains uncommon despite prolonged TKI exposure and highlight the need for therapeutic strategies specifically targeting stem-cell persistence [78]. Resistance—particularly in the context of complex mutational profiles—still limits outcomes in a subset of patients. In parallel, disparities in global access to both therapy and molecular monitoring underscore a critical unmet need, highlighting that the benefits of scientific progress are not yet equitably distributed.

Looking forward, the future of CML management will likely be shaped by three interrelated objectives: (i) increasing the proportion of patients achieving deep molecular responses and durable TFR, potentially through combination strategies and earlier use of novel agents such as asciminib; (ii) developing therapeutic approaches capable of targeting leukemic stem cells and overcoming resistance, including immunomodulatory and microenvironment-directed strategies; and (iii) ensuring global accessibility to effective therapies and standardized monitoring, thereby translating scientific advances into universal clinical benefit.

The history of CML demonstrates that transformative progress in cancer therapy is achievable when mechanistic understanding and therapeutic innovation evolve in parallel. As such, CML continues to serve not only as a model disease but also as a blueprint for the development of future targeted and potentially curative strategies across oncology.

## Figures and Tables

**Table 1:** **Comparison of BCR::ABL1 inhibitors.** CV, cardiovascular; GI, gastrointestinal; TKI, tyrosine kinase inhibitor.

Drug	Generation	Target Profile	Key Advantages	Major Toxicities	Clinical Notes
**Imatinib**	1st	ABL, KIT, PDGFR	Long-term safety, well tolerated	Edema, GI symptoms	Standard option, especially in older patients
**Dasatinib**	2nd	ABL + SRC	Potent, rapid responses	Pleural effusion	Avoid in pulmonary disease
**Nilotinib**	2nd	ABL (high affinity)	Deep molecular responses	Cardiovascular events	Avoid in high CV risk
**Bosutinib**	2nd	ABL + SRC	Favorable CV profile	Diarrhea, liver toxicity	Useful alternative
**Ponatinib**	3rd	ABL incl. T315I	Active in T315I	Arterial occlusive events	Use with dose optimization
**Asciminib**	Allosteric	Myristoyl pocket	Novel mechanism, well tolerated	Mild GI, fatigue	Option after ≥2 TKIs

**Table 2:** **ELN 2025—CML Chronic Phase Response Milestone.** ACAs, additional chromosomal abnormalities; CHR, complete hematologic response; CML, chronic myeloid leukemia; CyR, cytogenetic response; ELN, European LeukemiaNet; ELTS, EUTOS CML prognostic score; ^IS^, International Scale; MMR, major molecular response; MR, molecular response; Ph+, Philadelphia chromosome-positive; TKI, tyrosine kinase inhibitor.

Time Point	Optimal	Warning	Failure
**Baseline**	—	High ELTS risk score ACAs in Ph+ cells	—
**3 months**	BCR::ABL1 ^IS^ ≤ 10% (early molecular response)	BCR::ABL1 ^IS^ > 10% (interpret in context; not automatic switch)	No CHR achieved (hematologic resistance only)
**6 months**	BCR::ABL1 ^IS^ ≤ 1% (=partial CyR equivalent)	BCR::ABL1 ^IS^ > 1–10%	BCR::ABL1 ^IS^ >10% (confirmed)
**12 months**	BCR::ABL1 ^IS^ ≤ 0.1% (MMR/MR3)	BCR::ABL1 ^IS^ > 0.1–1% (individualize decision)	BCR::ABL1 ^IS^ >1% (resistance; individualize TKI switch)

**Table 3:** **Pivotal clinical trials evaluating TKI discontinuation and TFR outcomes.** 2G-TKI, second-generation tyrosine kinase inhibitor; DAS, dasatinib; DMR, deep molecular response; IMA, imatinib; MR, molecular response; MRec, molecular recurrence; NIL, nilotinib; TFR, treatment-free remission; TKI, tyrosine kinase inhibitor.

Trial	Discontinued TKI	Eligibility	TFR Rate	Key Findings
**STIM1**	IMA	MR5.0 ≥ 2 years	38% at 60 months	First prospective TFR study Duration of TKI treatment and Sokal Score Risk at diagnosis as predictors of MRec
**STOP 2G-TKI**	DAS/NIL	MR4.5 ≥ 2 years	54% at 48 months	Discontinuation of first/second line 2G-TKIs
**DASFREE**	DAS	MR4.5 ≥ 1 years	46% at 24 months	Duration of TKI treatment and age as predictors of MRec
**ENESTFreedom**	NIL	MR4.5 ≥ 1 years	43% at 5 years	Discontinuation of frontline NIL
**ENESTop**	NIL	MR4.5 ≥ 1 years	43% at 5 years	Discontinuation of second-line NIL
**EURO-SKI**	Any TKI	MR4.0 ≥ 1 years	46% at 36 months	Largest TKI discontinuation study Duration of TKI treatment and duration of DMR as predictors of MRec

**Table 4:** **Criteria and outcomes of treatment-free remission.** DMR, deep molecular response; LSC, leukemia stem cell; MMR, major molecular response; MR, molecular response; TKI, tyrosine kinase inhibitor, ^IS^, International Scale.

Parameter	Key Features
**Eligibility**	Chronic phase, ≥5 years TKI therapy sustained DMR (MR4.0/MR4.5) ≥2 years
**Monitoring**	Monthly for 6 months, then every 2–3 months
**Relapse definition**	Loss of MMR (BCR::ABL1 ^IS^ > 0.1%)
**Time to relapse**	Mostly within first 6 months after discontinuation
**Success rate**	~40–60% in selected patients
**Outcome after restart**	Rapid regain of response in most cases
**Predictors of success**	Longer TKI duration, deeper response, low risk
**Biological basis**	Immune control + LSC persistence

## Data Availability

No new data were created or analyzed in this study.
